# Sensitivity and Specificity of Developmental Surveillance and Autism Screening in an Australian Multicultural Cohort: The Watch Me Grow Study

**DOI:** 10.1007/s10803-025-06859-z

**Published:** 2025-05-15

**Authors:** Charmaine Bernie, Alicia Montgomery, Louise Sealy, Joseph Descallar, Cheryl Dissanayake, Bin Jalaludin, Elisabeth Murphy, Sue Woolfenden, Katrina Williams, Valsamma Eapen

**Affiliations:** 1 Southern Cross University, Gold Coast, QLD Australia; 2 Department of Paediatrics, Monash University, Melbourne, VIC Australia; 3 School of Population Health, University of New South Wales, Sydney, Australia; 4 Sydney Children’s Hospital, Sydney, NSW Australia; 5 Ingham Institute of Applied Medical Research, University of New South Wales, Liverpool, Australia; 6 Olga Tennison Autism Research Centre, School of Psychology and Public Health, La Trobe University, Bundoora, VIC Australia; 7 New South Wales Ministry of Health, Sydney, NSW Australia; 8 Sydney Institute of Women, Children and Their Families, Sydney Local Health District, New South Wales, Australia; 9 Sydney Medical School, University of Sydney, Camperdown, NSW Australia; 10 Monash Children’s Hospital, Melbourne, VIC Australia; 11 ICAMHS, L1 MHC, Liverpool Hospital, Elizabeth Street, Liverpool, NSW 2170 Australia

**Keywords:** Child development, Autism, Screening, Surveillance

## Abstract

To examine accuracy of two-tiered surveillance to detect developmental disability and autism in a multicultural birth cohort; a subset of the Watch Me Grow Study. Surveillance tools were used at or soon after 18 months of age, including the Parents’ Evaluation of Developmental Status (PEDS), the Ages and Stages Questionnaire (ASQ-3), and the revised Modified Checklist for Autism in Toddlers (M-CHAT-R/F). Children with and without identified concerns were assessed between 18 and 23 months using the Mullen Scales of Early Learning (MSEL) and the Autism Diagnostic Observation Schedule-2 (ADOS-2). Sensitivity and specificity of the surveillance tools used in isolation and in combination in this cohort ranged from 51 to 87% (n = 165). Some children (n = 21) who were not identified with high likelihood of difficulties were later assessed as having probable developmental disability. Adding the M-CHAT-R/F did not significantly improve autism likelihood identification in comparison with tiered developmental surveillance. There was highly variable sensitivity and specificity of combined tools for tiered developmental surveillance in this cohort. There remains a need in Australia to improve methods of, and engagement in, developmental screening and surveillance that includes detection of concerns in community and primary healthcare settings.

## Introduction

Developmental disabilities, including Autism Spectrum Disorder (ASD; herein described as autism) represent a health and economic burden (Olusanya et al., 2018; Solmi et al., 2022), with rising prevalence estimates (Carnemolla et al., 2020; Solmi et al., 2022). Despite improved outcomes for children identified and supported early, (Eapen et al., 2016; Kohli-Lynch et al., 2019), timely identification and intervention remains challenging (Anderson et al., 2017; Eapen et al., 2014; Gallegos et al., 2021).

Surveillance programs, typically including monitoring of development using specific tools or processes at repeated timepoints, can identify children with probable developmental concerns, facilitating access to assessment and supports (Barger et al., 2018; Lipkin et al., 2020). Given benefits of early supports (Kohli-Lynch et al., 2019; Nahmias et al., 2019), there is increasing emphasis on best practice identification of developmental concerns in the first two years of life (Barbaro et al., 2021; Hirai et al., 2018).

In Australia, services for children with developmental concerns are commonly funded within the National Disability Insurance Scheme (NDIS), with no requirement for a diagnosis to access functional supports (Arefadib, 2019; Purcal et al., 2018). In 2018, Australian diagnostic guidelines for autism was released recommending functional assessment and supports prior to diagnosis (Whitehouse et al., 2018). Though functional assessment principles and the need for early identification is well described, specific tools for screening and surveillance, and functional assessment, have not been prescribed at a national level either prior to or following implementation of the NDIS (Brewer et al., 2020). For children with unidentified support needs, improved and consistent surveillance systems are suggested, including those which adopt a tiered tool administration. (Barbaro et al., 2021; Eapen et al., 2017).

Surveillance may include one-off screening assessments at a population level (level 1 screening) followed by within group screening or surveillance for those with high likelihood for developmental concerns (level 2 screening) (Brewer et al., 2020). Despite support for ongoing surveillance, there are different approaches across Australia with regard to settings, the particular screening tools used and how often screening occurs (Brewer et al., 2020; Eapen et al., 2017; Mozolic-Staunton et al., 2020).

In some Australian states, the Parents’ Evaluation of Developmental Status or PEDS (Glascoe, 1999) is used for routine developmental surveillance (Edwards et al., 2020). This 10-item standardised screening tool provides parents with the opportunity to record concerns about their child’s development (Glascoe, 2006; Schonwald et al., 2009). The tool adds value during well-child appointments as recommended in children’s Personal Health Record (Çelen Yoldaş et al., 2021; Sheldrick et al., 2020). Health practitioner and parent discussions at the appointment help to clarify concerns and support access to needed assessments according to specific pathways (e.g. PEDS Paths A, B or C).

Previously, NSW Health recommended that children with identified developmental concerns on the PEDS are further assessed by a child health professional using a secondary (level 2) developmental screening tool, the Ages and Stages Questionnaire, Third Edition (ASQ-3) (Squires & Bricker, 2009) and Ages and Stages Questionnaire: Social-Emotional (ASQ:SE) (Squires et al., 2002). The ASQ-3 adds additional domain-specific skill acquisition details to support understanding of children’s developmental levels and subsequent decision-making.

The combination of PEDS and ASQ-3 administration as part of the developmental surveillance programme occurred up until 2017, when assessment of developmental concerns using the Centre for Disease Control’s Learn the Signs Act Early (Daniel et al., 2009), was introduced to replace the PEDS. There is inconsistency across Australia in relation to tools used to identify developmental concerns, recommended actions at level 1 or level 2 surveillance and screening, and follow up methods (Barbaro et al., 2021; McLean K, 2014).

While population-based developmental surveillance and screening data are widely available, tool accuracy in priority populations such as multicultural communities and/or those experiencing social disadvantage, or as part of a tiered screening process, has received less attention (Barger et al., 2018; Edwards et al., 2020; Hirai et al., 2018). Evaluation of tools and processes to detect developmental concerns at a secondary level is particularly important, given reports of lower sensitivity and specificity levels of well-known screening tools, such as the Modified Checklist for Autism (M-CHAT-R/F) (Robins et al., 2001) and the Social Communication Questionnaire (SCQ) (Rutter et al., 2003), when used clinically as a level 2 autism screener (Bernie et al., 2021; Haffner et al., 2021). The M-CHAT and its revised version (M-CHAT-R/F), inclusive of a parent interview have demonstrated large variability in specificity (38–99%) at this secondary screening level. (Matson et al., 2013; Petrocchi et al., 2020).

To address identified gaps, the “Watch Me Grow (WMG)” Study, previously described (Woolfenden et al., 2016), adopted a longitudinal prospective birth cohort design to evaluate the feasibility and accuracy of the developmental surveillance system for children in South West Sydney, NSW. This is an area of significant social disadvantage and cultural and linguistic diversity, with the sample being representative of the population (Woolfenden et al., 2016).

The capacity of the two-tiered surveillance system to detect developmental disability and autism was examined. The addition of an autism-specific screening tool, the M-CHAT-R/F, was evaluated in relation to later formal developmental and autism testing.

We include the accuracy of the PEDS, ASQ-3 and M-CHAT-R/F, which comprise the level 1 and level 2 screening tools used to identify likelihood of developmental delays and autism at approximately 18 months of age. We also report on the accuracy of the PEDS and M-CHAT-R/F alone and in combination with the ASQ-3 as part of the overall developmental surveillance system.

## Method

### Participants and Setting

Recruitment occurred between 2012 and 2013 from public hospital postnatal wards and child health nurses in NSW. Of the WMG Study Cohort (n = 2025), 1761 parent participants (87%) completed a baseline questionnaire following informed consent. Of those, a total of 565 (32%) went on to complete the PEDS, and an additional proportion of children completed the ASQ-3, and the M-CHAT R/F (Table 1). The mean age of the participants was 20 months (*SD* = 0.8 months).

**Table 1:** Sociodemographic details of participants and caregivers attending assessments

		Completed PEDS from WMG cohort (*N* = 565) n(%)	Assessments conducted (*N* = 166) n(%)	*χ*^*2*^* p* value or Fishers (Cramer’s V/phi co-efficient)	Tiered surveillance and assessment indicates developmental concern (*N* = 22) n (%)	Either surveillance or assessment indicates no concern (*N* = 144) n (%)	*χ*^*2*^* p* value or Fishers (Cramer’s V/phi co-efficient)
Child gender	Male	244 (44.9)	88 (53.0)	0.03*(0.1)	19 (86.3)	69 (47.2)	< 0.01* (0.3)
	Female		74 (44.6)		3 (13.6)	71 (49.3)	
	Unknown		4 (2.4)		–	4 (2.8)	
Mother's country of Birth	Australia	233 (42.9)	89 (53.6)	<0.01* (0.1)	7 (31.8)	82 (56.9)	0.02^*^(0.2)
	Overseas		75 (45.2)		15 (68.2)	60 (41.6)	
	Unknown		2 (1.2)			2 (1.4)	
Primary language	English		113 (68.1)		13 (59.0)	100 (69.4)	0.24 (0.1)
	Other	128 (23.6)	49 (29.5)	0.08 (0.1)	9 (40.9)	40 (27.8)	
	Unknown		4 (2.4)		–	4 (2.8)	
Mothers age at delivery (years)	≤ 20	8 (1.5)	4 (2.5)	0.48^c^	–	4 (2.9)	0.85 (0.1)
	21–29		71 (43.4)		9 (40.9)	62 (44.3)	
	30–39		80 (49.4)		12 (54.5)	68 (48.6)	
	≥ 40		7 (4.3)		1 (4.5)	6 (4.2)	
Mother’s education	No tertiary education		38 (22.9)		8 (36.4)	30 (20.8)	0.39 (0.1)
	Tertiary education^a^		124 (74.6)		14 (63.6)	110 (76.4)	
	Other		2 (1.2)		–	2 (1.4)	
	Unknown		2 (1.2)		–	2 (1.4)	
Annual household income^b^	< $25 000	60 (11.8)	17 (10.2)	1.0 (0.0)	5 (22.7)	12 (8.4)	0.16 (0.2)
	$25 000–$75 000		54 (32.5)		7 (31.8)	47 (32.6)	
	$75 000–$150 000		65 (39.1)		9 (40.9)	56 (38.9)	
	> $150 000		18 (10.8)		1 (4.5)	17 (11.8)	
	Unknown		12 (7.2)		–	12 (8.3)	

^a^including technical colleges

^b^All amounts in AUD

^c^Fisher’s Exact Test and no effect calculation completed due to cell count < 5

^*^p < 0.05

**Fig. 1:**
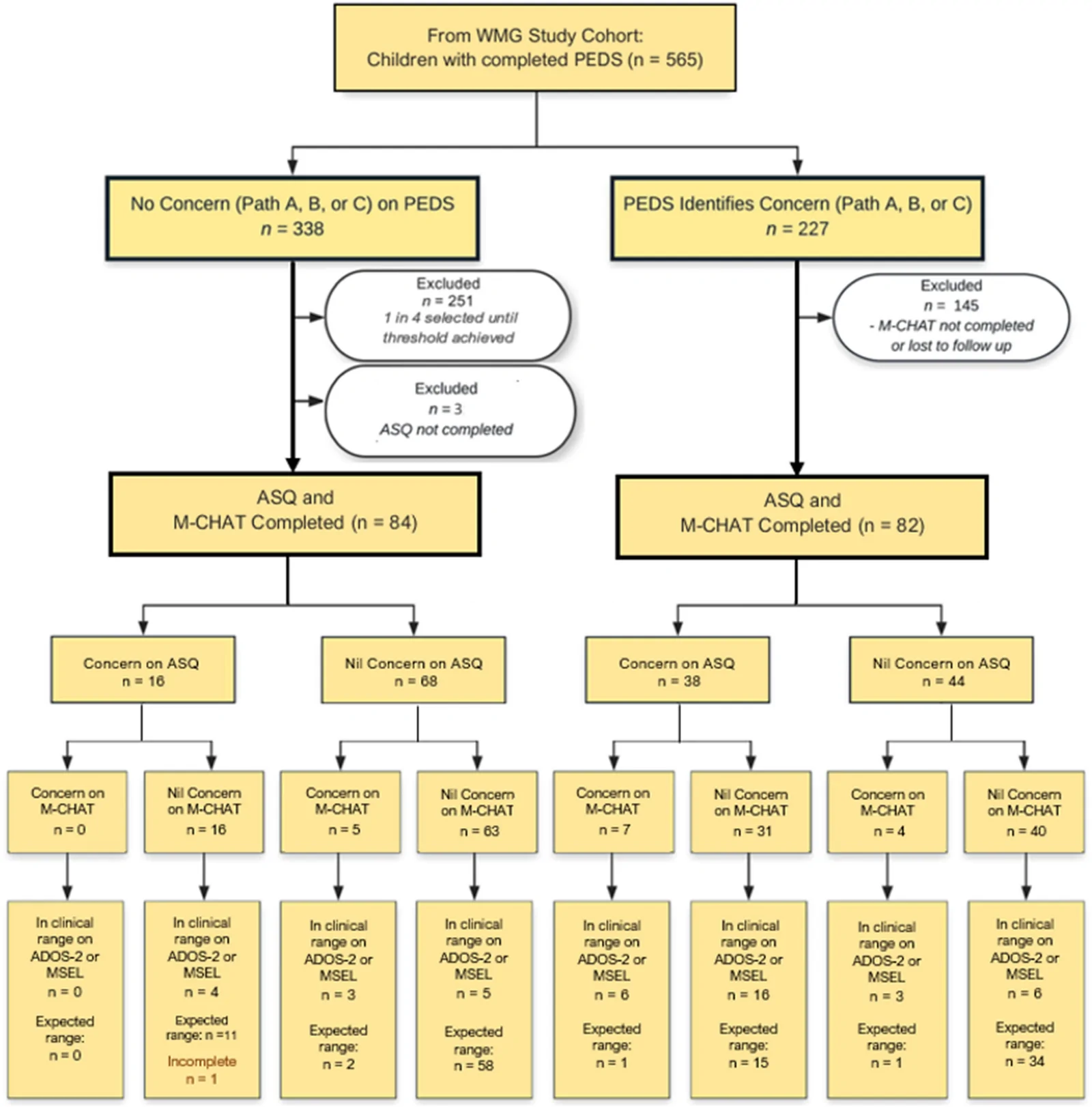
The Watch Me Grow (WMG) developmental surveillance study cohort and participant flow

Children who were identified on the PEDS as being at high likelihood for a developmental concern (n = 227) and every fourth child who was identified as having no developmental concern on the PEDS (n = 338) were invited to complete the M-CHAT-R/F. This process was adopted to allow for a sufficient number of participants from the “No Concern” group to proceed to the assessment phase and ensure a comparative sample that is adequately powered for comparison. Follow up assessments included the Mullen Scales of Early Learning (MSEL) (Mullen, 1995) and Autism Diagnostic Observation Schedule 2 (ADOS-2) (Lord, 2012). If a participant in the “No Concern” group did not respond, then the next child on the list was invited until the threshold was reached. Figure 1 shows the study flow diagram of participant inclusion.

For study purposes, a child was considered as having probable developmental disability following assessment if they scored ≥ 2 standard deviations below the mean on one or more cognitive subscales of the MSEL, and/or scored at ‘Mild-to-Moderate’ or ‘Moderate-to-Severe’ likelihood on the ADOS-2. There was a final sample of 166 participants with ASQ-3, M-CHAT-R/F, MSEL and ADOS-2 assessments conducted, with 165 complete data sets for sensitivity and specificity analysis.

The PEDS was completed with health professionals as part of a well-child check or via telephone with a trained researcher, with 72 of 166 (43%) completed in languages other than English (Arabic: *n* = 59; Vietnamese: *n* = 11; Urdu: *n* = 1; Bengali: *n* = 1), with the support of verified document translations and a service-supplied interpreter who read out questions where literacy issues were evident. Assessments were conducted in English in a child-friendly room with a familiar caregiver present.

### Ethics

The study was approved by the Human Research Ethics Committees of South Western Sydney Local Health District and the University of New South Wales (SWSLHD).

### Measures

#### Surveillance and Screening Measures

Surveillance and screening measures were selected in keeping with NSW Health’s statewide Personal Health Record program, which selected measures based on timings of well-child visits, capacity of health service providers and current evidence-based tools at the time of the study. (Woolfenden et al., 2016). The PEDS was used to identify developmental concerns from parental perspectives, across broad areas of development and behaviour, as part of the NSW Health Personal Health Record program. In the WMG study, developmental surveillance data was ascertained at six, 12 and 18 months. At 18 months, ≥ one concern in the global/cognitive, expressive language, receptive language and ‘other’ domains is considered indicative of developmental vulnerability. In earlier studies PEDS has demonstrated moderate to high sensitivity (74–97%) and moderate specificity (70–86%) (Glascoe, 2013; Warren et al., 2016; Woolfenden et al., 2014). Its psychometric properties have been found to be less robust among younger children ≤ 18 months of age with sensitivity range of 62% to 75% and specificity of 62% to 81% (Limbos & Joyce, 2011; Marlow et al., 2019).

The ASQ-3 and ASQ:SE are well-validated screening tools exploring communication, gross motor, fine motor, problem solving, adaptive, and social-emotional abilities. Parents complete the ASQ-3 independently, or if necessary with the assistance of a professional, by responding to a series of multiple-choice questions to help ascertain their child’s broad skill levels across each domain. The ASQ-3 has been found to have moderate to high sensitivity (70–90%) and specificity (76–91%) (Agarwal et al., 2020; Bricker et al., 1999; Sices et al., 2009; Squires et al., 1997). Fewer validation studies have been performed with the ASQ:SE, which is reported to have high sensitivity (81%) and specificity (84%) (Squires et al., 2015). Herein, only the ASQ-3 results were included in the analysis.

The M-CHAT-R/F is a 20 yes/no item screening tool for the early detection of ASD at 18 months (Robins et al., 2001, 2014), exploring social engagement, gestures and pretend play. The M-CHAT-R/F has moderate sensitivity (75–90%) and specificity (41–61%) across different populations (Yuen et al., 2018). Accuracy is variable when the tool is evaluated in developmentally-vulnerable groups, either at level 1 or level 2 (Petrocchi et al., 2020).

#### Assessment Measures

Assessment measures were selected based on commonly-adopted clinical assessment batteries in the region. These were typically selected based on evidence of assessment quality at the time of the study, and practicality of tool administration with the clinical populations involved (Eapen et al., 2013; Lord, 2012a, b). Due to study clinical and study resource limitations particularly in relation to completing detailed assessments with caregivers where English is a second language, recommended parent-interview tools such as the Vineland Adaptive Behaviour Scales for broader, detailed functional assessment were not included (Whitehouse et al., 2018).

The MSEL is a standardised measure of child development (ages up to 68 months), providing normative scores, where the mean is 50 (*SD* = 10) across five motor, language, and visual-reception subscales. The MSEL gives a composite score with a mean of 100 (*SD* = 15) and is common in research studies involving toddlers and young children, particularly when measuring developmental abilities of autistic children (Eapen et al., 2013; Lord et al., 2012a, b).The Early Learning Composite Score is derived from subtests not inclusive of the gross motor scale. It has an internal reliability value of 0.91 and adequate one to two-week test–retest reliability in the normative sample of infants and toddlers (0.82–0.96). There is also established convergent validity with the Bayley Scales of Infant Development is reported (Bayley, 1993).

The ADOS-2 is a semi-structured, standardised assessment commonly administered as part of an autism diagnostic process (Randall et al., 2018). The child’s social communication, behaviours and responses across activities are rated, with higher scores indicating greater likelihood of an autism diagnosis. Module 1 was used to assess relevant study participants in this subset, as the Toddler Module was not yet readily available in clinical settings in New South Wales at the time of study design and implementation. A community of practice was set up for all assessors ensuring fidelity and inter-rater reliability was maintained for research reliability. Participants from this study were utilised and the standard was set at equal to or above 80%.

### Data Analysis

Sensitivity and specificity of screening tools individually, and in combination, were calculated, with positive and negative likelihood ratios, and confidence intervals (95%CI) included. Sensitivity measures the ability of a screening tool to correctly identify individuals who have a condition (true positives). Specificity refers to the ability of a screening tool to correctly identify individuals who do not have the condition (true negatives). The MSEL and ADOS-2 were administered and scored by qualified clinicians, and the ADOS-2 was video-recorded for quality assurance and assessors’ own inter-rater reliability coding.

Data was analysed using Chi Square, Fisher’s Exact Test and Cramer’s V/phi coefficients via R 4.3.1 and online test calculators (http://araw.mede.uic.edu/cgi-bin/testcalc.pl), with the exception of sensitivity and specificity of combined tools. This was analysed using SAS Studio for Windows, Version 3.8 (SAS Institute., Cary, NC).

## Results

### Sample Demographics

The PEDS and M-CHAT-R/F were completed for 169 of 565 children (29.9%) invited to participate, 82 of whom screened PEDS Positive (49%), and 87 who screened PEDS Negative (51%). There were more children from the PEDS Negative (no concern) group who did not continue in the study as only a selected group were invited to continue (76% versus 63%), *X*^*2*^ (1, *N* = 169) = 6.98, *p* < 0.01, Φ = 0.1. Three participants who did not complete the ASQ-3 were also excluded from the analysis. In contrast to the sample of the WMG cohort that completed the PEDS, more children of Australian-born mothers participated in assessment, and a higher proportion of boys were included (Table 1).

The mean age of children who attended at follow up (*N* = 166) was 20.6 ± 0.8 months (range 18 to 23 months). No age difference for children with concerns (mean of 21.1, *SD* = 0.8) and without developmental concerns (mean of 21.2. *SD* = 0.8 months; *p* = 0.49) was found. Likewise, the mean age of mothers of children with and without developmental concerns was similar (mean of 31.2, *SD* = 5.1 years for both groups). Boys were more likely to present with developmental concerns (*p* < 0.01), as were children of mothers born overseas (*p* = 0.02).

### Sensitivity and Specificity of Developmental Surveillance and/or Autism Screening

Of the 165 children who completed assessment, 43 (26%) scored above clinical thresholds for either the ADOS or MSEL. Of these, 22 (13.3%) were identified as having probable developmental disability and had been identified from the tiered surveillance system. Five children had a high likelihood of autism on the ADOS-2 (22.7%), 15 (68.1%) had a developmental delay based on MSEL, and two had both (9%). A further 21 had been identified as having probable developmental disability without being identified on the tiered surveillance system described below (PEDS and ASQ-3). Table 2 details the accuracy of individual and tiered surveillance, based on the existing process in NSW and the addition of the M-CHAT –R/F, in relation to identification of probable developmental disability and autism likelihood in children assessed. Prior probability of scoring in the clinical range on assessment was 26%. One participant with incomplete data was excluded from analysis.

**Table 2:** Level 1 and level 2 surveillance and screening: sensitivity and specificity

Surveillance and screening type	N = 165^1^	Above threshold^2^	Below threshold^3^	Sensitivity [95%CI]	Specificity [95%CI]	Positive LR [95%CI]	Negative LR [95%CI]
Developmental surveillance (PEDS alone)	PEDS positive	31	51	72% [56, 85]	58% [49, 67]	1.7 [1.3, 2.3]	0.5 [0.3, 0.8]
	PEDS negative	12	71				
Tiered developmental surveillance (PEDS and ASQ-3)	PEDS and ASQ-3 positive	22	16	51% [35, 67]	87% [80, 92]	3.9 [2.3, 6.7]	0.6 [0.4, 0.8]
	PEDS and/or ASQ-3 negative	21	106				
Developmental surveillance and autism screening: PEDS and M-CHAT R/F	PEDS or M-CHAT-R/F positive, or both	34	53	79% [64, 90]	57% [47, 66]	1.8 [1.4, 2.4]	0.4 [0.2, 0.7]
	PEDS and M-CHAT negative	9	69				
Tiered developmental surveillance and autism screening: PEDS with ASQ-3 & M-CHAT-R/F combined	PEDS and ASQ-3, PEDS and M-CHAT-R/F or all tools positive	25	17	58% [42, 73]	86% [79, 92]	4.2 [2.5, 6.9]	0.5 [0.3, 0.7]
	PEDS negative, or PEDS positive with ASQ & M-CHAT both negative	18	105				

^1^Single participant excluded due to incomplete data

^2^Result on assessment indicates child’s performance in the clinical range for developmental disability/autism likelihood

^3^Result on assessment does not indicate child’s performance in the clinical range for developmental disability/autism likelihood

#### Tiered Developmental Surveillance (PEDS and the ASQ-3)

Adding the ASQ-3 to the PEDS (Row 2, Table 2) resulted in a decrease in sensitivity to 51%, 95% CI [35, 67] but an increase in specificity to 87%, 95% CI [80, 92] compared to PEDS alone (Row 1). The positive LR was 3.9, 95% CI [2.3, 6.7] and the negative LR was 0.6, 95% CI [0.4, 0.8]. There were no significant differences in the performance of the PEDS and the ASQ-3 in relation to children with mothers who did not speak English, were born outside of Australia, were younger, or part of households earning less than $75,000 per year.

#### Developmental Surveillance (PEDS) and Autism Screening (M-CHAT-R/F)

Based on the system of PEDS or M-CHAT-R/F (Row 3, Table 2), a sensitivity of 79%, 95% CI [64, 90], specificity of 57%, 95%CI [47, 66], a positive LR of 1.8, 95% CI [1.4, 2.4] and negative LR of 0.4, 95% CI [0.2, 0.7] was found. Here, 53 children were false positives and nine children who would have benefited from further assessment were missed.

#### Tiered Developmental Surveillance (PEDS and ASQ-3) and Autism Screening (M-CHAT-R/F)

The combined administration of PEDS and ASQ-3, with the M-CHAT R/F (final row, Table 2), had a sensitivity of 58%, 95% CI [42, 73] and a specificity 86%, 95% CI [79, 92]. This was a comparable performance to PEDS and ASQ-3 combined, with a sensitivity of 51% and a specificity of 87%. Subgroup analyses related to parent and child characteristics did not demonstrate significant differences in the accuracy of tools between groups.

## Discussion

The current study analysed the outcomes of different measures adopted as level 1 and tiered developmental surveillance approaches, with and without an autism-specific screening tool, in a subset of young, culturally diverse Australian children: the WMG Study cohort. In isolation, the PEDS as a level 1, standalone tool within a surveillance system in this sample had moderate sensitivity (72%) and low specificity (58%). These findings are broadly in line with recent studies examining the tool’s accuracy with younger children, albeit with slightly lower specificity found (Warren et al., 2016; Woolfenden et al., 2014), that is slightly greater chances of overidentifying children with probable developmental disability in this sample.

Based on the system of tiered developmental surveillance examined with this sample of children and families, the PEDS in combination with the ASQ-3 yielded mixed results with lower sensitivity (51%), but higher specificity (87%), compared with PEDS alone. That is, slightly greater chances in the combined system of having children with probable developmental disability missed in the tiered developmental surveillance system, compared with PEDS alone. With 21 children of the sample in the clinical range at assessment who were incorrectly identified by the PEDS or the ASQ-3 as having no developmental concerns, using single administrations of these combined tools to detect children in a sample such as this, with some who definitely need early intervention, remains problematic. Ongoing monitoring at 24 months may have occurred, but access to early supports could be delayed as a result. This result may have been related to the way families, particularly those with English as a second language, interpreted questions and provided responses. Given these results, it is suggested that developmental surveillance tools are administered at different time points until preschool, so more children with developmental concerns can be identified and supported to access services that can optimise short-term and long-term outcomes.

When the M-CHAT-R/F was added to the tiered surveillance system, children who were positive on the M-CHAT-R/F went through to further assessment as well as those scoring positively on both developmental surveillance tools. The sensitivity of this combined system was 58%, and specificity was 86%. These results were broadly similar to the tiered developmental surveillance system of PEDS and ASQ-3, though with slightly higher sensitivity and therefore three less children with probable developmental disability missed using this tool combination. The system would assess 17 children who were false positives and miss 18 children who would have benefited from further assessment.

In relation to autism detection, adding the M-CHAT-R/F as a screening tool only mildly increased the correct identification of children who would later score in the clinical range on the ADOS in this sample (58% versus 51%). This is in keeping with other studies that have found autism-specific screening using the M-CHAT-R/F at young age ranges, and in at risk populations, is not as accurate as screening in later preschool years, or as a level 1 screener administered at repeated time points (Petrocchi et al., 2020). Adding the M-CHAT-R/F to the PEDS as a combined level 1 surveillance and screener, mildly increased sensitivity in this sample (up to 79%), missing fewer children who would go on to score in the clinical range of the ADOS, but with a slight cost to specificity (down to 57%). For both PEDS alone and PEDS with M-CHAT, this specificity range can mean overidentification and increased burden on assessment services, or children and families on pathways that may not be appropriate. Such challenges are particularly problematic in regions with high numbers of vulnerable families, where there may be increased demand on public health services, and an elevated need for clear messaging about service needs for children, provided in multiple languages, and adaptations to minimise other barriers to accessing timely supports. Caution around its use as a one-off screener, in very young or at-risk populations, and with families from diverse backgrounds remains warranted.

It may be the case that younger ages at screening (around 18 months) and the cultural diversity in this cohort (30% with English as a second language and 45% of mothers born overseas) decreased the suitability and cultural appropriateness of tools in this sample. Given the loss of some families who did not complete the M-CHAT-R/F, and the small numbers of children with developmental concerns in this sample, it is challenging to draw firmer conclusions regarding accuracy of these tools in diverse populations. It is important to note that similar numbers of children with completed M-CHAT-R/Fs were yielded from both the PEDS positive (51%) and PEDS negative (49%) groups in this sample. Given issues with reductions in tool accuracy in younger ages, and with families from diverse cultural backgrounds as previously described, care should be taken when considering single time-point measures with similar groups (Sheldrick et al., 2020; Warren et al., 2016).

It is critical that further consideration is given to the development of screening and surveillance tools, for tiered delivery over time, that are valued by and appropriate for measuring developmental risk in children from culturally and linguistically diverse backgrounds. Considering limitations relating to generalisability from this study sample, it is also important for future studies to assess whether any cultural adaptations to existing tools could be considered, including for specific cultural groups, and conduct tool accuracy evaluations with a larger cohort where possible. This has occurred for children from Aboriginal and Torres Strait Islander backgrounds with tools such as the ASQ-TRAK (Simpson et al., 2021), but such work with other diverse groups is lacking.

The positive LRs ranged from 1.7 to 4.2 with wide CIs also noted, indicating further justification of caution around interpreting findings. Given the prior probability of 26%, these LRs do not provide a sufficient change to post-screening probability to inform decision-making about the need for further diagnostic assessment. It is imperative that further investigations continue into Level 1, and particularly Level 2 screening tools that can accurately identify developmental concerns with very young children. To date, research confirming the ability of screening tools to do this consistently well (at or above an 80% level) without high level input from clinicians has yielded variable findings (Barbaro et al., 2022; Petrocchi et al., 2020; Sheldrick et al., 2020).

Findings suggest that completing these tools alone, or in any combination before 2 years, do not detect children with developmental concerns with high accuracy. As with all screening tools there was a trade-off between sensitivity and specificity. It is also important to note that due to a substantial percentage of families in the PEDS positive arm who did not go on to complete ASQ-3 or M-CHAT-R/F screening tools, some bias in this clinical sample may exist. As findings are not out of keeping with other studies exploring PEDS sensitivity and specificity with younger age ranges, and the inclusion of similar numbers and demographics of PEDS negative children from the same birth cohort, there is likely to be sufficient internal validity and this risk is considered minimal. Given limitations and the lack of informative likelihood ratios, however, it is challenging for authors to suggest a leading surveillance tool combination in Australian community contexts, based on these results.

In addition to sample size limitations and a proportion of families who did not complete the additional screening tools, other study limitations include the study participants’ English speaking requirements, and lacking data on formal clinical diagnoses. Given a diagnosis was not confirmed by a clinical team as part of this study, results relating to probable developmental disorders should be interpreted cautiously.

Despite these limitations, some recommendations from these findings and directions for future research are suggested. Ongoing monitoring is needed in the early years, with clinician input, to maximise detection and ensure access to suitable supports. It is particularly important that this is done with culturally-appropriate tools and processes that acknowledge and address the needs of Australia’s multicultural families. This may include anticipatory and translated guidance for parents (Kohlhoff et al., 2022), ongoing and accessible opportunities for clinical assessment and discussions, and observations of child development and behaviour in natural environments, such as education and other community settings. Cross-departmental and cross-service monitoring systems, using culturally-appropriate, evidence-based tools and other additional monitoring processes, are suggested as an area of future research, coinciding with opportunistic contacts such as primary healthcare checks and immunisation visits to increase detection opportunities. These monitoring systems should also include children with familial, sociological, or environmental risk identified, such as children from families who are culturally and linguistically diverse and not initially identified as having developmental concerns, in keeping with study results and other investigations evaluating service inequities (Wallis et al., 2021). In combination with other surveillance and screening systems which use evidence-based tools over multiple time points, such strategies can ensure all children have repeated opportunities to access timely services that meet their needs in the early years and beyond.
